# Research progress on applications of calcium derived from marine organisms

**DOI:** 10.1038/s41598-020-75575-8

**Published:** 2020-10-28

**Authors:** Yangli Xu, Jian Ye, Deqing Zhou, Laijin Su

**Affiliations:** 1 Wenzhou Characteristic Food Resources Engineering and Technology Research Centre, Wenzhou Academy of Agricultural Sciences, Wenzhou, 325006 China; 2 Laboratory for Marine Drugs and Bioproducts of Qingdao National Laboratory for Marine Science and Technology, Yellow Sea Fisheries Research Institute, Chinese Academy of Fishery Sciences, Qingdao, 266071 China; 3 College of Life and Environmental Science, Wenzhou University, Wenzhou, 325035 China

**Keywords:** Nutrition, Natural products

## Abstract

Calcium is an important mineral that plays an integral role in human health, especially bone health. Marine biological calcium is an abundant resource that is generally accepted and has a complex active structure. This review evaluates research progress on marine biological calcium with regards to its sources, use of calcium supplements, calcium bioavailability, and novel applications of marine calcium. The potential for future development and the use of products incorporating marine biological calcium in biomedical research and the pharmaceutical, health care, and food industries are also reviewed. The goal of this review is to provide a comprehensive documentation on resource utilization and product development from marine organisms.

## Introduction

Calcium is an important micronutrient widely believed to affect bone health and human metabolism. Calcium deficiency can cause conditions like osteoporosis, rickets, epilepsy, and anemia. Calcium enters the circulation through food or calcium supplements, and a dynamic balance is maintained between blood and bone calcium^1^. The primary source of calcium is dairy products, including milk and its by-products like cheese and condensed milk, followed by other sources like cereals and tofu^2^. However, an inappropriate diet can decrease the bioavailability of calcium. For example, the presence of phytic acid in cereals and oxalic acid in green leafy vegetables can cause calcium to precipitate as calcium phytate and calcium oxalate, which are insoluble compounds^3^. In America, a study found that approximately 38% of adults who rely solely on food for mineral and vitamin intake consume inadequate levels of calcium, and approximately 93% consume inadequate levels of vitamin D, which plays a key role in calcium absorption rate, bone homeostasis, and bone repair^4,5^. Calcium deficiency becomes gradually debilitating with age^6^. Chronic calcium deficiency has caused osteoporosis to become an epidemic^7^. An increasing number of people continue to face calcium deficiency and diseases associated with calcium deficiency^8–10^. As a result, more people have increased their calcium intake through supplements based on the advice of doctors or the media^11^.

The calcium sources for these supplements include calcium carbonate ores, calcium-rich animal skeletons, marine shells, and crustaceans^12^. However, natural calcium carbonate ores may contain harmful elements, such as heavy metals^13^. Animal bones may carry the risk of prion transmission^14,15^. In recent years, calcium supplements from marine sources have gained attention due to their abundant reserves, high safety, and biological activity^16,17^. With the development and utilization of marine resources, more than 50% of fishery products, including bones, fins, heads, and internal organs, which are discarded as waste annually, can be used. Marine mineral supplements have the potential to increase bone turnover and may aid in preventing injuries and repairing damaged bone in humans^18^. As an abundant source of calcium, the use of marine biological calcium is an important way to improve the utilization rate of biological resources. This review comprehensively evaluates the marine calcium sources, the technology used for the preparation of calcium supplements, and the biological activity and bioavailability of marine calcium to provide references for the effective development of supplements using marine calcium.

## Marine source of calcium

**Figure 1:**
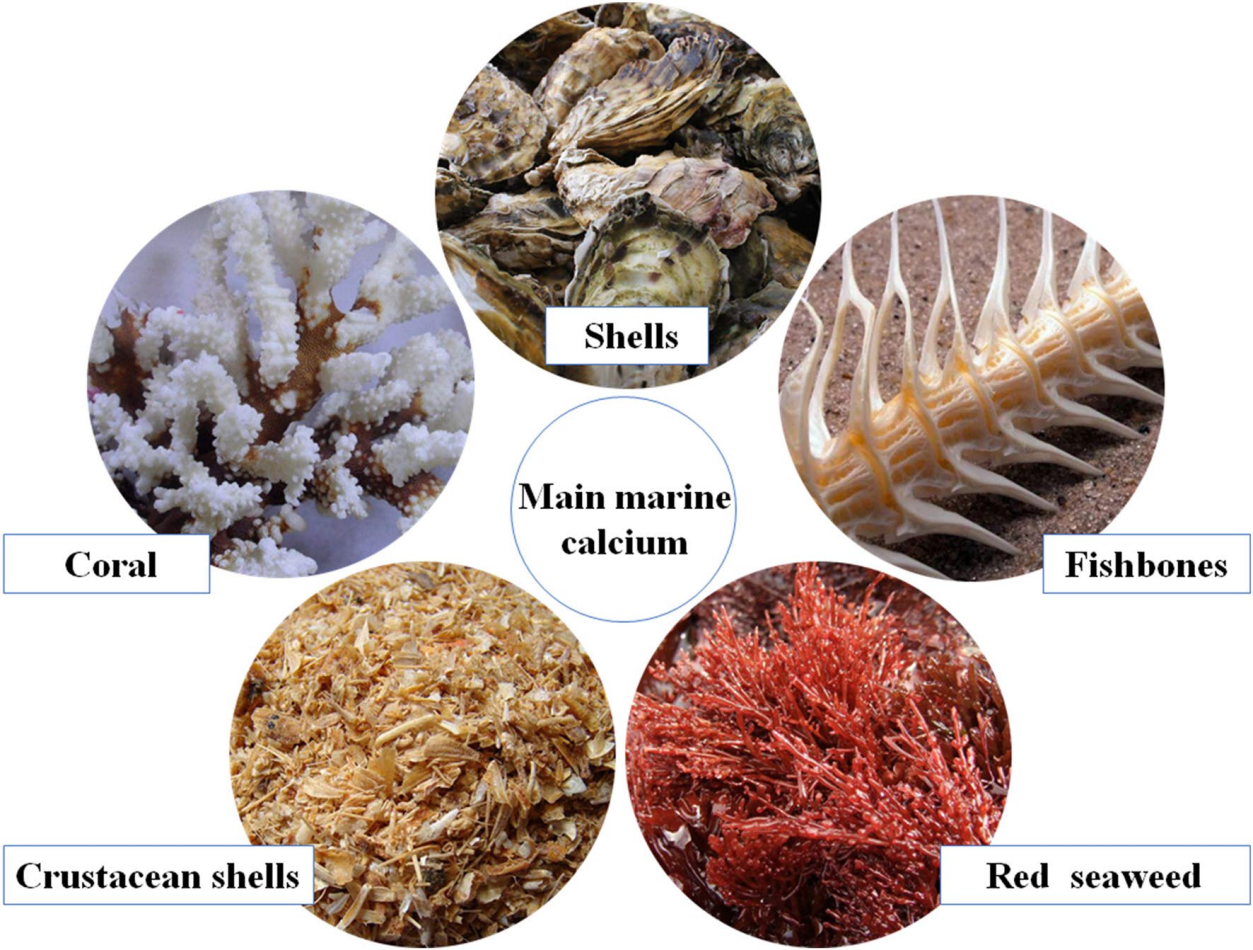
Main calcium source from Marine organism.

Oceans are rich in biological resources and calcium is an important mineral constituent of marine life. The major sources of calcium for humans from the oceans include fishbones, shellfish and crustacean shells, and coral and seaweed (Fig. 1).

### Calcium from fishbones

Fishbone is the general term encompassing the axial, appendage, and fishbone in the fish body, accounting for approximately 10–15% of the total body weight^19^. Fishbone tissue consists mainly of an organic extracellular matrix covered with hydroxyapatite [Ca_5_(PO_4_)_3_OH] and the calcium content found to be lowest in the salmonid species when compared to eight different species of fish was as high as 135–147 g/kg in the lipid-free dry matter^20^. Shark cartilage is another important source of calcium. For example, the calcium from the jaw cartilage of gummy shark is mainly in the form of hydroxy calcium phosphate crystal [Ca10(PO4)_6_(OH)_2_], and its calcium phosphate content is among the highest with 67% on a dry weight basis, which ranges between 124 and 258 g/kg^21^. Fishbones from large fishes need to be processed using chemical and biological methods to destroy organic material or bonded with collagen to increase the calcium dissolution rate because calcium in the form of hydroxyapatite is not suitable for absorption in humans^22^. Small fishes with soft bones, such as anchovies and lizardfish, can be processed into ready-to-eat food to be consumed with their bones^23^. Generally, calcium preparation from fishbones includes the removal of protein and fat by cooking, treating with alkali and organic solvents or enzymatic hydrolysis, and superfine crushing to obtain a fishbone powder.

### Calcium from shells

Shells account for approximately 60% of the mass of a shellfish, and calcium carbonate content in a shell can reach 95%. Shells are a rich source of high-quality marine calcium. Shellfish culture offers humans a low-impact source of sustainable protein^24^. In 2016, farmed shellfish reached 17.139 million tons globally, accounting for 21.42% of the total farm output^25^. Additionally, as the proportion of calcium in shells is higher than that in fishbones, the output from shells is greater^26,27^. The effects of calcium supplementation with Ezo giant scallop shell powder and fossil shellfish powder have been studied; the results indicated good solubility and bioavailability of calcium from these natural sources of calcium^28^. The shell calcium supplement was marketed in several countries worldwide; however, the utilization of shell resources remains low, and the comprehensive utilization and development of shell calcium require further support.

### Calcium from crustacean shells

People can directly ingest calcium by eating small dried shrimp or crabs. Crustacean processing and consumption generate 30–40% of marine resource waste^29^. Crustacean shells mainly comprise calcium carbonate (CaCO_3_), chitin, and protein^30^. Research on shrimp and crab shells have mainly focused on the utilization of chitin and protein resources, while calcium is sometimes recycled as a by-product, such as calcium hydrogen phosphate, calcium lactate, and calcium^31^.

### Calcium from coral

Coral calcium is formed from the exoskeleton of living organisms of many species^32^. Coral calcium is a natural source of marine calcium, containing 24% calcium, 12% magnesium, and more than 70 minerals; it has recently become a new international trend of calcium supplementation. Coral calcium is often used as a calcium supplement to treat bone metabolism disorders, osteoporosis, and other bone diseases^33,34^.

### Calcium from seaweed

Seaweed from the ocean, especially green algae, is rich in minerals such as calcium^35^. For example, Aquamin, a typical calcium-rich supplement derived from the calcified skeletal remains of the red seaweed species *Lithothamnion*, has calcium concentrations of up to 31%/weight^36^. A previous study has indicated that concerning calcium sources for horses, marine algae is better than calcium carbonate supplements^37^. Calcium extracted from marine algae was also found to show a beneficial anabolic effect on bone skeletal calcification in animal models of osteoporosis^38^. Algal calcium prepared from oyster shell powder and seaweed has a higher bioavailability than calcium carbonate^39^.

## Calcium supplements and bioavailability

### Direct ingestion of marine-derived calcium

The most common direct calcium supplements are small dried shrimp, shell powder, and small fishes. Several marine calcium supplements, such as oyster shells and coral calcium, have been commercialized in different countries. However, the main components derived from these marine sources are calcium carbonate and calcium polyhydroxy phosphate, which are difficult to absorb and increase gastric burden^40^. To improve the calcium absorption rate, marine sources are typically crushed or vacuum heated first^41,42^. Studies have found that marine-derived calcium has certain advantages over calcium carbonate supplements or other calcium-rich food. For example, Aquamin has better bioavailability and potential to slow down bone loss compared to calcium carbonate^36^. Hake fishbone (HBF) was a good source of calcium, with comparable efficacy to Lithotame (L), a calcium supplement derived from *Lithothamnion calcareum*^17^. A fishbone powder (Phoscalim) and a ray cartilage hydrolysate (Glycollagene) were comparable to milk for both short-term calcium absorption and bone resorption^16^. Tablets made with calcium from haddock bones were adequate for calcium supplementation and osteoporosis prevention^43^. Currently, the international recommended daily intake of calcium for general population is 700–1200 mg per day. However, teenagers (9–18 years old) need approximately 1300 mg calcium per day, and pregnant women with low dietary calcium intake need 1500–2000 mg calcium per day^44,45^. Studies have shown that more than 50% of the calcium deficient population include men and women older than 70 years, women aged 51–70 years, boys and girls aged 9–13 years, and girls aged 14–18 years^46^. Taking a conscious supplement of marine calcium is very effective in preventing calcium deficiency. Direct calcium ingestion from marine organisms is very suitable for daily calcium supplementation; however, it is insufficient for treating calcium deficiency diseases. In the treatment of diseases such as calcium deficiency, there is also a need to choose higher doses of calcium supplements or drugs^5^^.^

### Organic acid calcium

**Figure 2:**
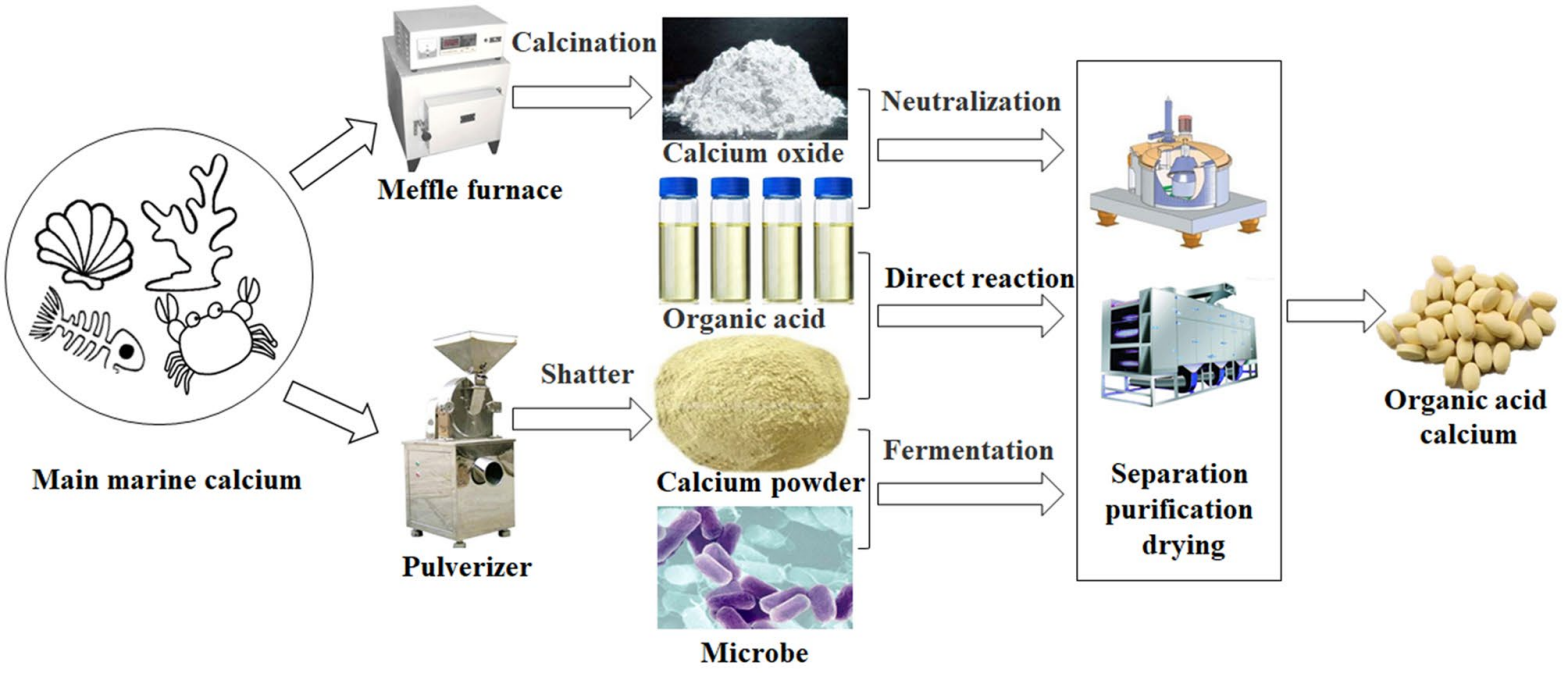
Flow chart of preparation of calcium organic acid from Marine calcium.

Organic acid calcium, such as calcium citrate, l-calcium lactate, calcium gluconate, calcium acetate, calcium formate, and calcium propionate, have higher bioavailability, solubility, and absorption rates, regardless of gastric contents, because they are less sensitive to gastric pH than calcium carbonate^11,40,47^. It is mainly prepared by neutralization or fermentation of calcium compounds (Fig. 2). As a dietary calcium supplement, calcium formate has been found to exhibit significant advantages over both calcium carbonate and calcium citrate^48^. Calcium glucoheptonate has exhibited a high relative bioavailability of calcium and is well-tolerated in humans than calcium carbonate^49^. However, calcium gluconate and calcium lactate are less concentrated forms of calcium, making them impractical oral supplements. Calcium acetate and calcium propionate are not widely used either^50^. Calcium organic acids alone are not good for absorption because they can bind to oxalic acid or phytic acid present in food. Calcium combined two or more organic acids, such as calcium citrate malate (CCM)^10^, which combines bovine collagen peptides with calcium citrate^51,52^. The combined use of polycan and calcium lactate–gluconate^53,54^ was found to have beneficial synergistic effects compared with the use of calcium organic acids alone.

Marine sources of calcium organic acids are primarily fishbones, shrimp, crab shells, and other shells^3^. To facilitate easy calcium absorption, appropriate processes such as calcination, enzymatic hydrolysis and fermentation methods should be selected according to the nutritional composition and associated processing properties^55,56^. Subsequently, citric acid, gluconic acid, lactic acid, acetic acid, and/or propionic acid are added to prepare calcium organic acids. The solubility and bioavailability of calcium from natural sources of shellfish calcium with citrate and lactate were increased after decompression treatment^26^. Fishbones can be fermented with *Leuconostoc mesenteroides* to obtain high amounts of soluble calcium with free calcium, calcium amino acids, calcium acetate, small peptide calcium, and calcium lactate. The fermentation of grass fishbones can increase calcium bioavailability and also help avoid wastage of fishbone calcium and aquatic proteins^57^.

### Calcium chelate

**Figure 3:**
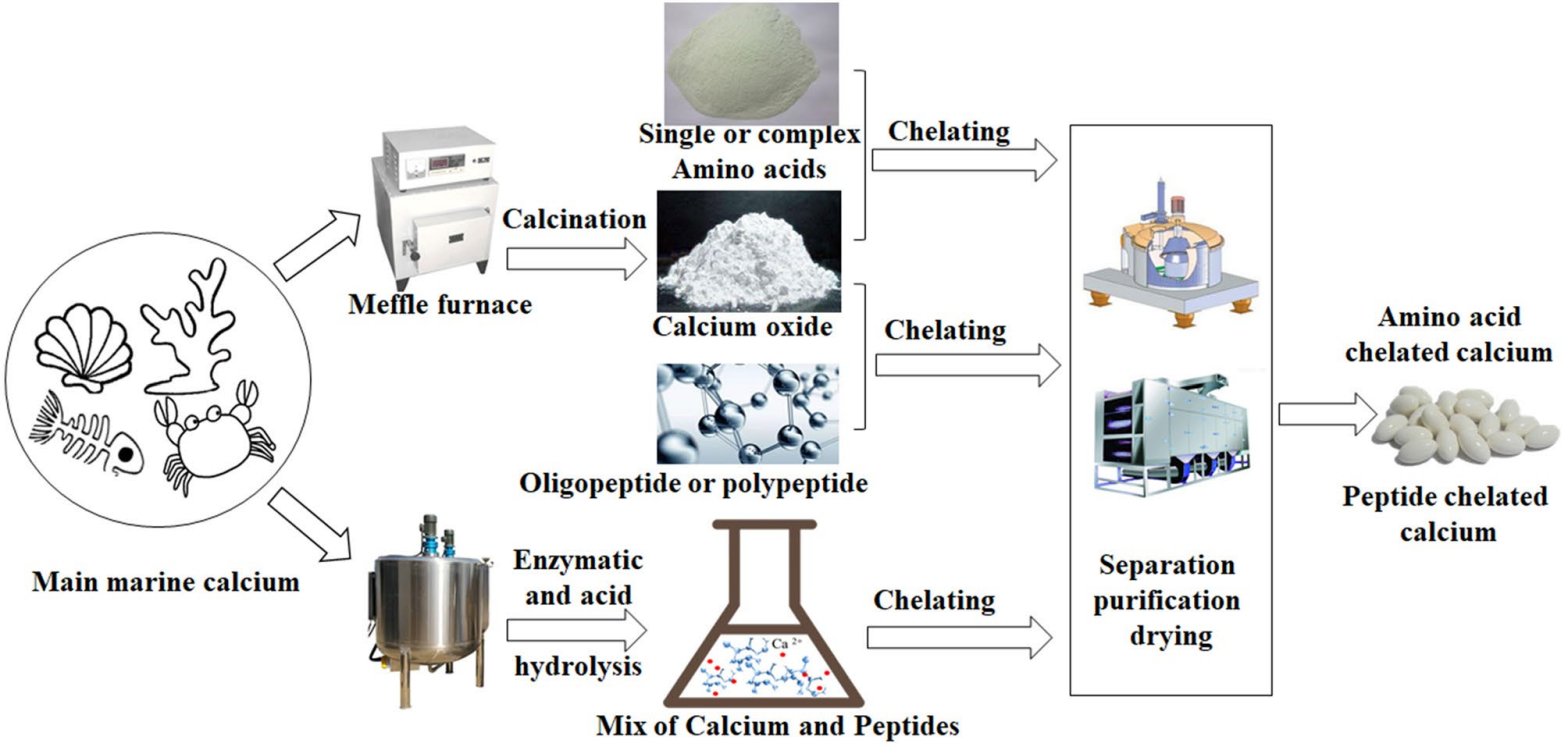
Flow chart of preparation of chelate calcium from Marine calcium.

Calcium chelate refers to the metal complex formed by stable bonds between amino acids or peptides and metal calcium ions and includes two main products, calcium amino acid and calcium peptide chelate^58–60^. It is mainly prepared by chelating polypeptides or oligopeptides with calcium ions or when a single or complex amino acid chelates with calcium ions (Fig. 3). Amino acid chelated calcium is not dependent on vitamin D3 and can be absorbed by the human body through amino acid metabolism. For example, calcium lysinate, a new form of calcium preparation, may have better absorption, making it a better calcium supplement than calcium carbonate and CCM^61^. However, peptide chelated calcium has advantages over other calcium supplements^62–64^. A growing number of chelating peptides have been identified and have been shown to promote and improve mineral bioavailability^65,66^. The calcium peptide chelate produced by combining fishbone calcium and calcium-binding bone collagen peptide through enzymatic hydrolysis demonstrated improvement in calcium bioavailability^67–69^. The algae peptide-based calcium-chelating complex and calcium alginate nanoparticles have the potential to be utilized as a calcium supplement to improve bone health^70–72^. However, the production cost of peptide chelated calcium is high, and the yield is low. With the development of new preparation technology, peptide chelated calcium will likely become a good calcium supplement.

## Other functions of marine source calcium

### Biological activity

Marine biological calcium has biological functions other than improving calcium homeostasis and bone health. For example, coral calcium was shown to regulate blood pressure and prevent the metastasis of colon cancer^30,73,74^. Calcium spirulan, derived from Spirulina platensis (*Arthrospira platensis*), a filamentous blue-green microalga from rivers and lakes, was shown to inhibit herpes simplex virus 1 actively, and possibly, infections caused by other herpesviruses^75^. Coral calcium hydroxide can act as an antioxidant, slowing senescence in mice and preventing hepatic steatosis^76–78^. The calcium oxide made from scallop shells was shown to inhibit *Pseudomonas aeruginosa,* a spoilage bacterium for eggs with a strong resistance to chemical agents such as sanitizers and disinfectants^79^. Calcium derived from oysters exhibited good efficacy in suppressing the formation and proliferation of oral squamous cell carcinoma^80^.

### New materials

Calcium from marine sources can serve as a raw material for the production of high-value-added compounds that can be used in biomedical research and pharmaceutical, healthcare, and food industries^81^. Previous studies have found a huge potential for producing porous scaffolds from oyster shells, clamshells, cuttlefish bones, and salmon bones^82–85^. The structural features of these scaffolds were found to be conducive to improve biological activities, including mechanical properties, and bone tissue growth and vascularization^86^. The production of natural hydroxyapatite (nHAP) from salmon bones and rainbow trout has a great potential as bone implant material substitutes in bone tissue engineering^87^. Marine biological calcium can also be used to prepare adsorption materials, demonstrating its potentially wide applications in water treatment. For example, calcium-rich biochar prepared from crab shells can be used to remove dyes and phosphorus from wastewater^88,89^. The acid-insoluble calcium silicate hydrates synthesized from oyster shells were also applicable in removing organic pollutants and heavy metal ions^90^. Single-phase hydroxyapatite (HA) and biphasic calcium phosphate (HA/β-TCP), which are derived from Atlantic cod bones, have no known cytotoxic effects and have demonstrated good bioactivity in simulated body fluid^91^. Consequently, calcium phosphate derived from marine organisms has a promising future in fabricating bacterial infection-resistant bone substitutes or bone defect healing. HA (Ca_10_(PO_4_)_6_(OH)_2_, HAp) derived from codfish bones is a calcium phosphate, which is a safer option for sunscreen formulation, indicating its potential across a wide range of applications in health care products and cosmetics^92^.

### Food additives

Biological calcium from marine processing waste can still be used in food processing. For example, fish bones can be added to fish surimi to improve the gel performance of the product^93^. Oyster shell calcium powder can improve the chewiness and springiness of restructured ham^94^. Calcium-rich shrimp and crab shells can also be used to prepare food flocculants^95^. There are many food additives containing calcium, such as calcium carbonate, calcium silicate, calcium sulphate, and calcium lactate. The calcium additives from marine organisms may be safer because they have a natural origin.

## Conclusions and future perspectives

Marine processing waste is often considered useless; however, it is an abundant and low-cost source of calcium. A study found that 55 brands of calcium supplements can be classified into seven categories based on the major ingredient in them and three or more categories were found to be derived from marine organisms mainly oyster/clamshells, algae, shark cartilage, and chelated calcium products (Table 1)^10^. In addition, calcium from marine organisms has good bioavailability and biological function. Reusing by-products from marine organisms can increase the added calcium value and reduce the risk of environmental pollution. For the development of calcium supplements, future work should focus on the comprehensive utilization of proteins, collagen, chitin, calcium, and other nutrients in marine organisms and the use of specific active ingredients to increase the bioavailability of calcium. In other applications, research must likely focus on the transformation of marine calcium into health foods, new materials, or food additives to expand to a commercial scale.

**Table 1:** Common commercial calcium supplements from Marine sources.

Name	Brand	Country	Calcium form	Source
Calcium carbonate	Life	Canada	Oyster shell powder, VD3	Oyster shells
Calcian + D3	Jamieson	Canada	Calcium citrate, calcium malate, fumaric acid calcium, calcium succinate, calcium carbonate, VD3	Oyster shells
Natural calcium	FOR BECARED ONE	USA	VD3, calcium, collagen type II	Oyster shells
Shen Gu Pian	Duo Yuan Kang	China	Oyster shell powder	Oyster shells
MC calcium	MC	Japan	Oyster shell powder, ursodeoxycholic acid, lysine hydrochloride	Oyster shells
Coral calcium capsules	Catalo	USA	100% pure coral powder	Coral
Coral calcium	Holland and Barrett	UK	100% pure coral powder	Coral
Haibrusk Shark cartilage	Bjorge ocean	Norway	Shark cartilage powder	Shark cartilage
Bonecare Kids calcium complex chews	Clinicians	New Zealand	Calcified lithothamnion, alcarerum-red algae, trimagnesium citrate, boron citrate, zinc amino acid chelate, manganese amino acid chelate, VD3	Red algae
Atomy tri-active calcium	Atomy	Korea	Seaweed meal, magnesium oxide, calcium citrate, serum calcium, VD	Red algae
